# Comparison of quality of life in patients with advanced chronic kidney disease undergoing haemodialysis, peritoneal dialysis and conservative management in Johannesburg, South Africa: a cross-sectional, descriptive study

**DOI:** 10.1186/s40359-023-01196-1

**Published:** 2023-05-08

**Authors:** Neelu Mathew, Malcolm Davies, Feroza Kaldine, Zaheera Cassimjee

**Affiliations:** 1 University of the Witwatersrand, Johannesburg, South Africa; 2 Division of Nephrology, Helen Joseph Hospital, Johannesburg, South Africa; 3 Department of Psychology, Helen Joseph Hospital, Johannesburg, South Africa

**Keywords:** Health related quality of life, Haemodialysis, Peritoneal dialysis, Chronic kidney disease

## Abstract

**Introduction:**

Mental health and quality of life are under-appreciated clinical targets which affect patient and modality survival. Lack of dialysis availability in the resource-constrained public health sector in South Africa results in assignment to treatment modalities without regard to effects on these parameters. We assessed the effect of dialysis modality, demographic and laboratory parameters on mental health and quality of life measurements.

**Methods:**

Size-matched cohorts were recruited from patients on haemodialysis (HD), peritoneal dialysis (PD), and patients on conservative management (CM) between September 2020 and March 2021. Responses to the Hospital Anxiety and Depression Scale (HADS) and Kidney Disease Quality of Life Short Form 36 (KDQOL-SF36) questionnaires and demographic and baseline laboratory parameters were compared between modalities. Multivariate linear regression was used to evaluate independent effect of baseline characteristics on HADS and KDQOL-SF36 scores between treatment groups where significant difference was observed.

**Results:**

Anxiety, depression, and reduced KDQOL measures were widespread amongst respondents. Dialyzed patients reported higher anxiety and depression scores than those on CM (p = 0.040 and p = 0.028). Physical composite (PCS), role–physical (RP), vitality (VS), and emotional well-being (EWB) KDQOL-SF36 scores were poorer in dialyzed patients (p < 0.001 for all). PCS (p = 0.005), pain (p = 0.030), vitality (p = 0.005), and social functioning KDQOL scores were poorer in PD compared to HD; HADS anxiety (p < 0.001) and KDQOL-SF36 EWB scores (p < 0.001) were better in PD. PD patients were more likely to be employed (p = 0.008). Increasing haemoglobin concentration reduced anxiety (p < 0.001) and depression scores (p = 0.004), and improved PCS (p < 0.001), and pain scores (p < 0.001). Higher serum albumin improved PCS (p < 0.001) and vitality (p < 0.001) scores.

**Conclusion:**

Advanced chronic kidney disease increases anxiety and depression and limits quality of life. PD improves mental health and emotional wellbeing and preserves the ability to undertake economic activity but limits social functioning and causes greater physical discomfort. Targeting haemoglobin may ameliorate modality effects on mental health and quality of life.

**Supplementary Information:**

The online version contains supplementary material available at 10.1186/s40359-023-01196-1.

## Introduction

A significant burden of communicable and non-communicable diseases, combined with poor access to nephrology services, render chronic kidney disease (CKD) not uncommon in low-to-middle income countries (LMIC); in Africa genetic risks exacerbate this pattern [1, 2]. South Africans are at increased risk of CKD due to a combination of these genetic and environmental risk factors [1]. Socioeconomic improvements since the advent of democracy are likely to exacerbate this risk as urbanization and lifestyle change increase the prevalence of hypertension and diabetes [2].

Although the majority of South Africans are reliant on the public sector for management of kidney failure (KF), long-term under-resourcing of state-sponsored dialysis units has resulted in disparities in access to treatment, with the dialysis treatment rate in the private sector being 787 per million population (pmp) as compared to 57 pmp in the state sector [3]. Patients with KF in the state sector may therefore not be able to access dialysis and may instead receive conservative management. Those patients fortunate enough to receive dialysis often do not have the luxury of choosing a preferred modality; instead, many state facilities pursue a policy of “peritoneal dialysis first”. Lack of participation in treatment planning may adversely affect treatment satisfaction and quality of life (QOL) [4]. Increased survival rates on dialysis, combined with declining rates of transplantation in South Africa, [5] contribute to prolonged waitlisting, in turn leading to accrual of aging and dialysis vintage-associated pathologies which may compromise QOL. In addition, dialysis treatments themselves carry the potential for significant morbidity which may offset improvements in QOL attained through amelioration of uraemic symptoms.

QOL is known to affect future risk of hospitalisation and mortality [6, 7], as well as compliance with therapy [8]. In addition, QOL is itself a significant outcome measurement of kidney replacement therapy (KRT) [9]. Despite this, QOL remains an underappreciated and under-researched component of KF management, and there is a paucity of data on the quality of life experienced by dialysis patients in the South African context [10–12]. In particular, the effect of non-directed dialytic therapy on QOL has not been contextualised against the QOL of patients receiving conservative management for KF. This study compares the quality of life of patients on peritoneal and haemodialysis against those with advanced CKD not receiving dialysis and considers the effect of comorbidities on quality-of-life scores.

## Methods

### Study design and settings

Helen Joseph Hospital (HJH) is a tertiary-level facility which provides specialised nephrology services to residents of the western areas of Johannesburg. The dialysis unit at HJH has the capacity to provide haemodialysis to 74 patients and peritoneal dialysis to 80 patients. Entrance onto the dialysis programme is regulated by local policy in consideration of transplant eligibility. Patients excluded from dialysis are referred to the Renal Outpatients Department for further treatment. Size-matched cohorts (n = 50, reflecting the minimum number of patients attending dialysis clinics) were voluntarily recruited from the outpatient haemodialysis, peritoneal dialysis, and Renal Outpatients Department at HJH using convenience sampling between September 2020 and March 2021. Non-dialyzed patients were only considered for inclusion if baseline eGFR was consistently below 20 mL/min/1.73 m^2^ for at least 3 months. Patients with a history of hospitalization within the antecedent month were excluded.

## Study instruments

Participants were administered the Hospital Anxiety and Depression Scale (HADS) and the Kidney Disease Quality of Life Short-Form 36 (KDQOL-SF36) questionnaires during routine clinical appointments. Participants who were unable to complete the questionnaires independently or who required translation of the questionnaires into their vernacular language were assisted by nursing staff or the primary investigator. KDQOL-SF36 responses were transformed to domain scores using the online RAND health group KDQOL-SF^TM^ 1.3 calculator. Respondent demographics, HADS and transformed KDQOL-SF36 domain scores were entered with most recent laboratory results in an anonymized database which was used for statistical analysis using Statistica version 14.0.1 (TIBCO Software, Palo Alto, California).

Both the HADS and the KDQOL-SF36 surveys are validated for use in patients with advanced CKD [13, 14]. The KDQOL-SF36 consists of 36 questions which provide information on quality of life across a number of subdomains, including physical functioning, role-physical, pain, vitality, emotional well-being, role-emotional, and social functioning; scoring is from 0 to 100 with higher scores indicating better function. Physical function and mental health subdomain scores are used to calculate composite physical and mental score, reflective of overall limitation in these respective areas. The HADS scale questionnaire produces a score of 0–21 for both anxiety and depression symptoms, a score of more than 8 correlates with overt clinical disorder, whilst a score of 4–8 is indicative of borderline symptomatology.

### Statistical analysis

Distribution of continuous variables was analysed through Shapiro Wilk W testing and visual inspection of the histogram plot. Baseline laboratory and demographic characteristics were compared between treatment groups using ANOVA and Pearson Chi-square testing as indicated; the Kruskall Wallis ANOVA and Mann Whitney U test were used for non-parametric continuous data. HADS and KDQOL-SF36 scores were compared between treatment groups using the ANOVA, Student t-, Kruskall Wallis ANOVA, or Mann–Whitney U test as indicated. Where statistically significant difference was observed between treatment groups for quality-of-life measurement, subsequent analysis using multivariate stepwise sigma-restricted linear regression was undertaken to test the independence of treatment modality from confounding effect using a model comprising a priori factors (ethnicity, sex, source of income, and relationship status) and factors showing variation between treatment modalities (age, comorbid diabetes, dialysis vintage, haemoglobin, phosphate and albumin concentration, and parathyroid hormone level); significant multicollinearity of parameters was excluded with all parameters having a variance inflation factor below 2 and tolerance above 0.5 (Additional file 1: Table S1).

### Ethics approval

Approval for this study was obtained from the Human Research Ethics Committee of the University of the Witwatersrand (protocol number M200635). Institutional approval was obtained from Helen Joseph Hospital. The study was conducted in accordance with the principles of the Declaration of Helsinki.

## Results

One hundred and fifty patient participants were recruited to this study, comprising 50 patients on haemodialysis (HD) and peritoneal dialysis (PD) each, and 50 patients with advanced kidney dysfunction (eGFR less than 20 mL/min/1.73 m^2^) undergoing conservative outpatient management (CM). Amongst CM participants, 31 (62%) were in stage 5 of CKD (eGFR consistently below 15 mL/min/1.73 m^2^). Baseline characteristics are shown in Table 1 below.

**Table 1:** Baseline characteristics of participants

	Haemodialysis (n = 50)	Peritoneal dialysis (n = 50)	Conservative management (n = 50)	Conservative management stage 5 (n = 31)
Age (years)	45.5 ± 12.3	44.1 ± 11.6	59.0 ± 13.5	59.9 ± 14.1
Sex	Male: 23 (46%)	Male: 25 (50%)	Male: 27 (54%)	Male: 14 (45.2%)
	Female: 27 (54%)	Female: 25 (50%)	Female: 23 (46%)	Female: 17 (54.8%)
Race	African: 37 (75%)	African: 34 (68%)	African: 33 (66%)	African: 19 (61.1%)
	Asian: 3 (6%)	Asian: 2 (4%)	Asian: 0 (0%)	Asian: 2 (6.5%)
	Mixed race: 9 (18%)	Mixed race: 8 (16%) Caucasian:	Mixed race: 14 (28%)	Mixed race: 6 (19.4%)
	Caucasian: 1 (2%)	6(12%)	Caucasian: 3 (6%)	Caucasian: 4 (12.9%)
Relationship status	Married: 14 (28%)	Married: 19 (38%)	Married: 21 (42%)	Married: 10 (32.3%)
	Partner: 9 (18%)	Partner: 5 (10%)	Partner: 3 (6%)	Partner: 2 (6.4%)
	None: 27 (54%)	None: 26 (52%)	None: 26 (52%)	None: 19 (61.3%)
Source of income	Unemployed: 47 (94%)	Unemployed: 42 (84%)	Unemployed: 38 (76%)	Unemployed: 25 (80.6%)
	Social grant: 37 (74%)	Social grant: 21 (42%)	Social grant: 29 (58%)	Social grant: 17 (54.8%)
Dialysis vintage (months)	47 [15–82]	24 [13–40]	–	–
Access in HD patients / PD modality	Tunneled cuffed catheter: 31 (62%)	CAPD: 45 (90%)	–	–
	Arteriovenous Fistula: 14 (28%)	CCPD: 5 (10%)		
	Arteriovenous graft: 5(10%)			
Comorbidities	Diabetic: 11 (22%)	Diabetic: 8 (16%)	Diabetic: 25 (50%)	Diabetic: 14 (45.2%)
	HIV positive: 11 (22%)	HIV positive: 10 (20%)	HIV positive: 11 (22%)	HIV positive: 7 (22.6%)
	Cardiovascular disease: 48 (96%)	Cardiovascular disease: 43 (86%)	Cardiovascular disease: 47 (94%)	Cardiovascular disease: 31 (100%)
Hemoglobin (g/dL)	9.5 [8.7–10.0]	10.9 [9.3–12.6]	11.3 [9.6–12.8]	11.1 [9.4–12.8]
rEPO dose (units/month)	40,000 [24,000–64,000]	32,000 [0–48,000]	–*	–
Urea (mmol/L)	19.0 [11.8–26.1]	20.8 [15.9–25.5]	20.1 [15.8–27.3]	21.6 [16.9–30.5]
Calcium (mmol/L)	2.22 [2.13–2.40]	2.20 [2.09–2.30]	2.27 [2.14–2.37]	2.28 [2.10–2.36]
Phosphate (mmol/L)	1.71 [1.26–2.40]	1.76 [1.15–2.6])	1.38 [1.07–1.66]	1.48 [1.20–1.72]
Albumin (g/L)	40 [36–42]	35 [30–38]	40 [37–44]	40.0 [37–44]
Parathyroid hormone (ng/mL)	34.4 [14.8–59.8]	48.2 [29.6–79]	15.1 [8.7–21.5]	18.4 [10.8–21.5]

Values are mean ± SD or median [interquartile range]

*Patients on CM are precluded from rEPO prescription by local resource constraints

Significant differences in age (p < 0.001), haemoglobin (p = 0.007), phosphate (p = 0.002) concentration, parathyroid hormone (PTH) (p < 0.001), and albumin concentration (p < 0.001) were detected between the CM and dialysis treatment groups; diabetes mellitus was more frequent in patients on the CM programme than those receiving dialysis (p < 0.001). Haemoglobin concentration was higher (p = 0.001) and PTH control better (p = 0.027) in patients prescribed PD compared to those receiving HD; albumin concentration was, however, poorer in the former (p < 0.001). Dialysis vintage was greater in patients receiving HD compared to those on PD (p = 0.017).

Sex, level of education, relationship status, prevalence of HIV infection and cardiovascular disease, recombinant erythropoietin (rEPO) dose, and serum urea and calcium showed no significant difference between groups; prevalence of diabetes was similar between patients prescribed PD and those prescribed HD (p = 0.611). No significant difference was detected for age between the PD and HD cohorts (p = 0.494).

Unemployment was more frequent in the HD treatment group (p = 0.044). Patients on PD were less frequently supported by a social grant than other treatment groups (p = 0.008), whilst patients on HD were more frequent recipients of a social grant than other groups (p = 0.005).

**Fig. 1:**
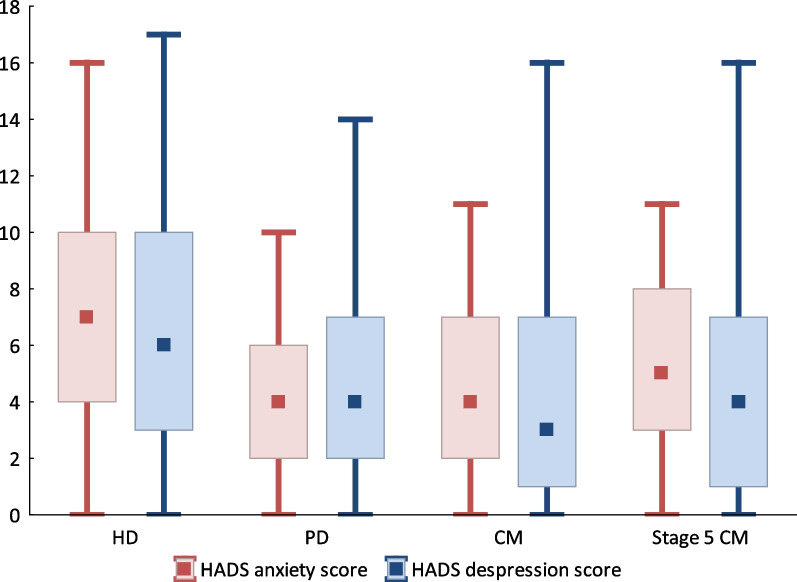
HADS anxiety and depression scores between treatment groups

Features of anxiety and depression were widespread amongst all respondents (Fig. 1, Table 2). Anxiety symptoms of significance (HADS anxiety score greater than 4) were significantly more frequent in the HD group (22, 44%) than in the PD (8, 16%) or CM (11, 22%) groups (p = 0.004). HADS anxiety score was higher in patients on dialysis compared to those on CM (p = 0.040), although this difference was ameliorated with progression to CKD stage 5 in the latter group (p = 0.284). Patients receiving HD reported higher anxiety scores than either those receiving PD (p < 0.001) or those on CM with CKD stage 5 (p = 0.045).

**Table 2:** HADS and KDQOL-SF36 scores across treatment groups

	All dialysis	HD	PD	CM	CM stage 5
*Hospital anxiety and depression scale*					
Anxiety	5 [3–8]	7 [4–10]	4 [2–6]	4 [2–7]	5 [3–8]
Depression	5 [2 – 8.5]	6 [3–10]	4 [2–7]	3 [1–7]	4 [1–7]
*Kidney disease quality of life—short form 36*					
Physical composite (PCS)	60.2 ± 10.7	63.2 ± 9.6	57.3 ± 10.9	66.2 ± 7.7	64.9 ± 8.4
Physical functioning (PF)	49.4 ± 10.4	48.7 ± 11.6	50.0 ± 9.0	51.5 ± 10.3	51.5 ± 10.1
Role – physical (RP)	58 ± 35.3	60 ± 35.4	56.0 ± 35.6	82.5 ± 26.8	79.0 ± 29.6
Pain (PS)	81.5 ± 21.2	86.1 ± 16.7	76.9 ± 24.3	85.8 ± 17.0	84.0 ± 17.2
General health (GH)	54.2 ± 10.6	54.4 ± 10.6	53.9 ± 10.6	50.1 ± 9.3	51.3 ± 9.6
Mental composite (MCS)	51.1 ± 8.4	53.6 ± 9.3	48.6 ± 6.6	50.7 ± 7.6	51.2 ± 7.5
Vitality (VS)	60.3 ± 10.7	63.2 ± 9.6	57.3 ± 10.9	66.2 ± 7.7	64.9 ± 8.4
Social functioning (SF)	54.5 ± 18.9	59.3 ± 17.8	49.8 ± 18.9	55.8 ± 14.8	53.6 ± 15.5
Role – emotional (RE)	40.7 ± 3.7	46.7 ± 32.3	34.7 ± 32.3	32.0 ± 27.7	36.6 ± 27.7
Emotional well-being (EWB)	47.7 ± 12.3	40.4 ± 10.0	55.0 ± 9.8	54.6 ± 7.5	55.3 ± 7.8

Values are median [interquartile range] or mean ± SD

Thirty-four percent (17 respondents) in the HD treatment group reported symptoms suggestive of depression (HADS depression score greater than 4) compared to 24% (12 respondents) in the PD treatment group and 22% (11 respondents) of those on CM (p = 0.348). HADS depression score was higher in patients on dialysis compared to those receiving CM (p = 0.028) although this significance was lost when analysis was restricted to the CM group with CKD stage 5 (p = 0.664). Depression scores were higher in patients prescribed HD, although statistically significant difference was not shown in comparison to those receiving PD (p = 0.092) or CKD stage 5 patients on CM (p = 0.124).

**Fig. 2:**
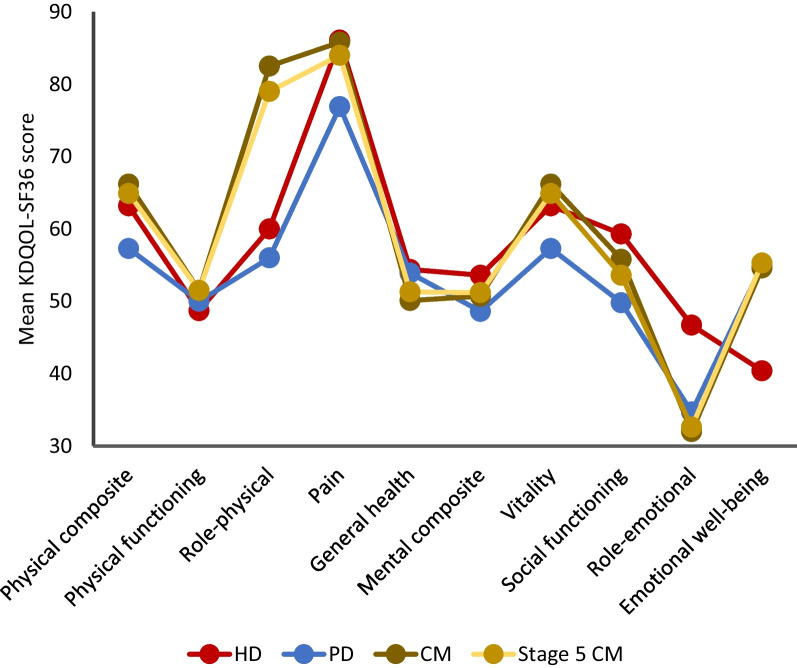
KDQOL-SF36 scores between treatment groups

Patients receiving either dialysis modality reported poorer KDQOL-SF36 physical composite score (PCS) than those on CM (p < 0.001), a finding which persisted when analysis was restricted to those CM patients with CKD stage 5 (p = 0.027) (Table 2, Fig. 2). Subjective assessment of ability to meet expected physical activity (role–physical, RP) was similarly poorer in patients on dialysis compared to these CM groups (p < 0.001 and p = 0.003, respectively). Dialyzed patients additionally reported lower vitality (VS) (p < 0.001 and p = 0.027, respectively) and emotional well-being (EWB) (p < 0.001 and p = 0.002, respectively) compared to CM groups. No significant differences were detected between patients receiving dialysis and those on conservative management for the domains of physical functioning (p = 0.232), pain (p = 0.212), general health (p = 0.181), mental composite score (p = 0.960), social functioning (p = 0.683), or role-emotional (p = 0.110).

Amongst dialysis recipients, PCS was significantly lower in those on PD compared to those prescribed HD (p = 0.005). Patients receiving PD additionally reported a lower mental composite score (MCS) compared to those prescribed HD (p = 0.003). Pain scores (PS) were poorer in patients prescribed PD compared to those receiving HD (p = 0.030). Vitality scores were lower in patients receiving PD compared to those on HD (p = 0.005) Social functioning score (SF) was similarly poorer in patients on PD compared to those on HD (p = 0.011); EWB was however better in the PD cohort compared to those on HD (p < 0.001). No significant difference was observed between dialysis modalities for the subdomains of physical function (p = 0.533), RP (p = 0.574), general health (p = 0.814), or role-emotional (p = 0.066).

Taken together, these findings suggest poorer quality of life amongst patients receiving dialysis across measurements of composite physical health (PCS), self-assessed ability to meet expectations of physical activity (RP), subjective feelings of fatigue (VS), and emotional well-being (EWB). In addition, a possible effect for prescribed dialysis modality was observed for HADS anxiety score, PCS, pain (PS), general mental health (MCS), VS, and EWB.

Stepwise sigma-restricted multivariate linear regression modelling was used to evaluate the confounding effect of baseline characteristics on scores which showed significant difference between treatment modalities (Tables 3, 4, 5).

**Table 3:** Effect of baseline characteristics on HADS anxiety and depression scores

	HADS anxiety score		HADS depression score	
	β ± SE β	p	β ± SE β	p
*Patients living with advanced chronic kidney disease*				
	R^2^adj 0.18, p < 0.001		R^2^adj 0.08, p < 0.001	
Age	− 0.21 ± 0.08	0.008	–	–
Black African ethnicity	− 0.19 ± 0.07	0.012	–	–
Relationship status	− 0.17 ± 0.07	0.021	–	–
Income source	–	–	− 0.19 ± 0.08	0.015
Haemoglobin	− 0.27 ± 0.07	< 0.001	− 0.23 ± 0.08	0.004
*Patients receiving dialysis*				
	R^2^adj 0.22, p < 0.001			
Relationship status	− 0.22 ± 0.09	0.018		
Prescribed peritoneal dialysis	− 0.20 ± 0.09	0.037		
Haemoglobin	− 0.31 ± 0.10	0.001

**Table 4:** Effect of baseline characteristics on KDQOL-SF36 physical health domains

	Physical composite		Role–physical		Pain	
	β ± SE β	p	β ± SE β	p	β ± SE β	p
*Patients living with advanced chronic kidney disease*						
	R^2^adj 0.12, p < 0.001		R^2^adj 0.14, p < 0.001			
Black African ethnicity	0.18 ± 0.08	0.024	–	–		
Income source	0.17 ± 0.08	0.029	–	–		
On dialysis programme	–	–	− 0.29 ± 0.08	< 0.001		
Haemoglobin	–	–	0.21 ± 0.08	0.008		
Albumin	0.27 ± 0.08	< 0.001	–	–		
*Patients receiving dialysis*						
	R^2^adj 0.16, p < 0.001				R^2^adj 0.24, p < 0.001	
Black African ethnicity	–	–			0.39 ± 0.09	< 0.001
Prescribed peritoneal dialysis	− 0.40 ± 0.10	< 0.001			− 0.30 ± 0.09	0.002
Haemoglobin	0.34 ± 0.10	< 0.001			0.32 ± 0.09	< 0.001

**Table 5:** Effect of baseline characteristics on KDQOL-SF36 mental health domains

	Mental composite		Vitality		Social functioning		Emotional well-being	
	β ± SE β	p	β ± SE β	p	β ± SE β	p	β ± SE β	p
*Patients living with advanced chronic kidney disease*								
			R^2^ 0.12, p < 0.001				R^2^ 0.22, p < 0.001	
Black African ethnicity			0.18 ± 0.08	0.023			–	–
Source of income			0.17 ± 0.08	0.029			− 0.15 ± 0.07	0.041
On dialysis programme			–	–			− 0.44 ± 0.08	< 0.001
Albumin			0.27 ± 0.08	< 0.001			− 0.39 ± 0.08	< 0.001
*Patients receiving dialysis*								
	R^2^adj 0.10, p < 0.001		R^2^adj 0.16, p < 0.001		R^2^adj 0.09, p = 0.003		R^2^adj 0.40, p < 0.001	
Black African ethnicity	–	–	–	–	0.22 ± 0.10	0.022	–	–
Source of income	–	–	–	–	–	–	− 0.16 ± 0.08	0.046
Peritoneal dialysis	–	–	− 0.40 ± 0.10	< 0.001	− 0.23 ± 0.10	0.017	0.51 ± 0.08	< 0.001
Haemoglobin	− 0.33 ± 0.0	< 0.001	0.34 ± 0.10	< 0.001			–	–
Albumin	–	–	–	–	–	–	− 0.23 ± 0.08	0.008

Improving haemoglobin concentration was associated with an ameliorated anxiety and lower depression scores across all treatment groups. Long-term relationships appeared to exert a salutatory effect on anxiety, with the effect being more significant for patients on dialysis. In this group, prescription of PD in preference to HD further reduced anxiety. Younger age was associated with increased anxiety across the cohorts as a whole.

Acceptance onto dialysis was associated with reduced perceived ability to perform expected physical activities (RP) and poorer emotional well-being. Improving haemoglobin concentration was associated with better role-physical score; higher albumin concentration was associated with better PCS and vitality scores, but poorer EWB. In patients receiving dialysis, prescription of PD was associated with poorer PCS, worse pain score, lower vitality, and poorer social functioning; the modality was, however, associated with better EWB. Haemoglobin concentration in dialyzed patients showed positive associations with PCS, pain, and vitality scores; an inverse association was observed for MCS. Black African ethnicity was associated with better reported pain and social functioning scores. Improving albumin concentration showed negative association with EWB. Age, patient sex, relationship status, comorbidity with diabetes, dialysis vintage, and phosphate and parathyroid hormone concentration showed no association with any of the analysed domains.

## Discussion

This study emphasises the significant psychosocial challenges faced by patients living with advanced chronic kidney disease. Underpinning disordered psychological health, patients experience significant physical limitation resulting in distress regarding their ability to meet expected levels of physical function. Whilst disease-dependent physiological factors such as anaemia may contribute to these symptoms, choice of dialysis modality may play an important role in the severity of depression and anxiety and in individual emotional well-being.

Population studies estimate the prevalence of depression amongst South Africans to be 9.8% and that of anxiety disorder to be 15.8% [15]; prevalence of symptoms of these disorders as evidenced by HADS scoring was higher in this cohort of patients living with advanced CKD. Prevalence rates of depression and anxiety disorders have been reported to be higher in patients living with CKD than in either the general population [16–18] or in patients living with other chronic diseases [17], and to increase with progression of chronic kidney disease stage [19].

HRQOL scores deteriorate with advancing CKD as uraemic symptoms mount [20, 21]. Physical function is prominently affected by CKD due to the limitations imposed on activity by ensuing anaemia, fluid retention, and acidosis [21]. Reflecting this, physical functioning was reduced in all treatment groups in this study. Consistent with the high prevalence of mood and anxiety disorders amongst patients in this study, emotional well-being was generally poor in all treatment groups, and patient subjective assessment of ability to meet expected emotional function (role–emotion) was uniformly poor in all respondents. Amelioration of the physical symptoms of uraemia by kidney replacement therapies may not necessarily bring about parallel improvements in emotional wellbeing, as evidenced by reductions in this subdomain in patients receiving dialysis compared to those assigned to conservative management in the present study. Loss of body autonomy, dependency on KRT for survival, fear of future therapy-related complications, and the constant threat of imminent mortality have been identified as significant contributors to emotional distress in patients receiving chronic dialysis [22].

Experience of these therapy-related stressors in the context of age-determined expectations of health may partially explain the inverse association between anxiety scores and age observed in this and other studies [23]. Younger patients may exhibit poorer emotion coping skills, in turn contributing to greater psychological distress and poorer quality of life [24].

Patient participation in the KRT planning process may ameliorate fears around therapy and restore a degree of autonomy, in turn improving satisfaction with treatment and quality of life [4]. Proponents of PD further cite improved patient satisfaction with the modality arising from reduced impact of dialysis treatments on quality of life [22]. In South Africa, where resource constraints restrict patient choice of dialysis modality, this reported benefit of PD is often used to justify the institution of “PD-first” programmes. Actual evidence for any difference in mental health and quality of life between dialysis modalities depends on few studies with contradictory findings [17, 25, 26].

Apparent differences in quality-of-life measurements between dialysis modalities may in part reflect confounding effects of therapy-related factors. For example, selection for PD requires home circumstances of a higher socioeconomic status, a known factor in quality-of-life scores [27]. PD patients in this study were more likely to be employed than those on HD and were less frequent recipients of social grants than other treatment groups, evidencing better socio-economic circumstances in this group. Haemoglobin concentration, which is known to affect both physical [11, 20] and mental [20, 21] quality-of-life scores, was lower in HD patients, reflecting a host of modality-related factors including blood loss in the extracorporeal circuit and vascular access procedures. Patients prescribed PD may manifest lower albumin concentration due to protein loss in dialysate effluent [28]; albumin has in some studies been associated with physical quality of life scores [29, 30].

Consistent with previous studies [11, 20, 21], higher haemoglobin concentration in the present study was independently associated with better physical quality of life, vitality, and reduced anxiety scores. Improved tissue oxygenation as haemoglobin levels rise is a probable contributor to physical quality of life and vitality measures; amelioration of anaemia-related symptoms may similarly account for improvements in reported anxiety. Albumin has traditionally been viewed as a marker for nutritional status in patients living with advanced CKD, although factors such as inflammation and fluid overload may also affect serum concentrations [28]. Such clinical correlates likely underlie the association between albumin and physical composite and vitality quality-of-life scores observed in this study, which are broadly consistent with previous findings reported by other researchers [29–31]. Interestingly, in the present study albumin demonstrated an inverse correlation with emotional well-being. Improvements in physical health and fatigue associated with increased albumin concentration may exacerbate discontent with the limitations imposed on daily living by the diagnosis of advanced chronic kidney disease, leading to lower emotional well-being. Further study is, however, required to test this hypothesis.

In this study, prescription of PD was independently associated with reduced anxiety and improved emotional well-being. Metanalysis has suggested some improvement in emotional distress and psychological well-being measures for patients on PD compared those on in-centre HD [32]. Additionally, PD recipients were more likely to maintain financial functionality, a finding which is consistent with other studies [33]. Despite these salutatory effects, overall composite mental score was lower in PD patients in the present study than in patients on HD, reflecting the contribution of lower-scoring domains such as social functioning, vitality, and the related subjective assessment of emotional role fulfilment (role–emotional) to the composite mental score. Reduced social functioning and vitality in patients receiving PD has been reported in other studies [33] and reflect the continuous nature of the modality with the need to perform frequent exchanges and associated disordered sleep [34]. Poorer physical composite score in the PD cohort of the present study is likely to have been influenced by the significantly poorer pain score reported by these patients. Abdominal discomfort engendered by frequent dialysate indwells [34] in patients receiving PD is likely to have resulted in a higher pain score in this group relative to those receiving intermittent HD.

### Limitations

There are limitations to the present study. Treatment groups were not well matched for age, dialysis vintage, and comorbidities, and had significant differences in a number of measured laboratory parameters. Although not measured, there is also likely to have been significant variation in eGFR between treatment groups, as well as Kt/V urea clearance between the HD and PD cohorts. The systematics of provision of dialytic therapy in the local context, and the effect of different treatments on lab parameters, make these differences unavoidable, and their distribution in this study is reflective of clinical reality. Limitations imposed by the cross-sectional nature of this study should also be acknowledged. Although the exclusion of recently admitted patients and the administration of the questionnaires on routine appointment days were employed to limit the effects of recent perturbations on responses, it may be that long-term pooled data would be better reflective of the burden of mental health disorders in these patients. Finally, the single-centre nature of this study may limit the generalizability of the findings.

## Conclusions

The present study confirms the significant prevalence of anxiety, depression, and reduced quality- of-life in patients living with advanced CKD, but also offers hope. In particular, the direct effect of PD on reducing anxiety and improving emotional well-being, and the potential indirect effect of PD on mental health through facilitation of employment, provides reassurance on the “PD-first” programmes adopted by many state units in South Africa and other LMIC. Deleterious effects of CKD on quality-of-life may be ameliorated through attention to haemoglobin targets, and through nutritional interventions to improve albumin. Younger patients receiving KRT may particularly require additional mental health support to cope with the demands of treatment.

## Supplementary Information

**Additional file 1**. Multicollinearity analysis for included regression parameters

**Additional file 2**. Hospital Anxiety and Depression Scale

**Additional file 3**. KDQOL-36 Survey

## Data Availability

Data is available on request from the corresponding author.

## Abbreviations

Alb: Albumin
ANOVA: Analysis of Variance
CAPD: Continuous Ambulatory Peritoneal Dialysis
CKD: Chronic Kidney Disease
CM: Conservative Management
eGFR: Estimated Glomerular Filtration Rate
HADS: Hospital Anxiety and Depression Scale
Hb: Haemoglobin
HD: Haemodialysis
HR-QOL: Health Related Quality of Life
IQR: Interquartile Range
KDIGO: Kidney Disease Improving Global Outcome
KDOQI: Kidney Disease Outcome Quality Initiative
KDQOL-SF: Kidney Disease Quality of Life-Short Form
KF: Kidney Failure
LTRRT: Long Term Renal Replacement Therapy
MWUT: Mann Whitney U Test
PD: Peritoneal Dialysis
Pmp: Per Million Population
PO4: Phosphate
PTH: Parathyroid Hormone
ROPD: Renal Out Patient Department
RRT: Renal Replacement Therapy
SD: Standard Deviation
SF-36: Short Form-36
QOL: Quality of Life
WHO: World Health Organisation
X^2^: Chi Squared

## References

[1] Naicker S (2010). Burden of end-stage renal disease in sub-saharan Africa. Clin Nephrol.

[2] George C, Mogueo A, Okpechi I, Echouffo-Tcheugui JB, Kengne AP (2017). Chronic kidney disease in low-income to middle-income countries: the case for increased screening. BMJ Glob Health.

[3] Davids MR, Jardine T, Marais N, Sebastian S, Davids T, Jacobs JC (2021). South African Renal Registry report 2019. Afr J Nephrol.

[4] Callahan MB (2001). Using quality of life measurement to enhance interdisciplinary collaboration. Adv Renal Replace Ther.

[5] Moosa MR (2019). The state of kidney transplantation in South Africa. S Afr J Med.

[6] Mapes DL, Lopes AA, Satayathum S, Mccullough KP, Goodkin DA, Locatelli F, Fukuhara S, Young EW, Kurokawa K, Saito A, Bommer J, Wolfe RA, Held PJ, Port FK (2003). Health-related quality of life as a predictor of mortality and hospitalization: the Dialysis Outcomes and Practice Patterns Study (DOPPS). Kidney Int.

[7] Oshus TBH, Preljevic VT, Sandvik L, Leivestad T, Nordhus IH, Dammen T, Os I (2012). Mortality and health-related quality of life in prevalent dialysis patients: comparison between 12-items and 36-items short-form health survey. Health Qual Life Outcomes.

[8] Nagasawa H, Tachi T, Sugita I, Esaki H, Yoshida A, Kanematsu Y, Noguchi Y, Kobayashi Y, Ichikawa E, Tsuchiya T, Teramachi H (2018). The effect of quality of life on medication compliance among dialysis patients. Front Pharmacol.

[9] Lee MB, Bargman JM (2016). Survival by dialysis modality—who cares?. Clin J Am Soc Nephrol.

[10] Seedat YK, MacIntosh CG, Subban JV (1987). Quality of life for patients in an end-stage renal disease programme. S Afr Med J.

[11] Okpechi IG, Nthithe T, Swanepoel CR (2013). Health-related quality of life in patients on haemodialysis and peritoneal dialysis. Saudi J Kidney Dis.

[12] Tannor EK, Archer E, Kapembwa K, van Schalkwyk SC, Davids MR (2017). Quality of life in patients on chronic dialysis in South Africa: a comparative mixed methods study. BMC Nephrol.

[13] Loosman WL, Siegert CEH, Korzec A, Honig A (2010). Validity of the Hospital Anxiety and Depression Scale and the Beck Depression Inventory for use in end-stage renal disease patients. Br J Clin Psychol.

[14] Aiyegbusi OL, Kyte D, Cockwell P, Marshall T, Gheorghe A, Keeley T, Gheorghe A, Keeley T, Slade A, Calvert M (2017). Measurement properties of patient-reported outcome measures (PROMs) used in adult patients with chronic kidney disease : a systematic review. PLoS ONE.

[15] Herman AA, Stein DJ, Seedat S, Heeringa SG, Moomal H, Williams DR (2009). The South African stress and health (SASH) study: 12-month and lifetime prevalence of common mental disorders. S Afr Med J.

[16] Murtagh FEM, Addington-hall J, Higginson IJ (2007). The prevalence of symptoms in end-stage renal disease: a systematic review. Adv Chronic Kidney Dis.

[17] Goh ZS, Griva K (2018). Anxiety and depression in patients with end-stage renal disease : impact and management challenges—a narrative review. Int J Renovasc Dis.

[18] Zalai D, Szeifert L, Novak M (2012). Psychological distress and depression in patients with chronic kidney disease. Semin Dial.

[19] Palmer S, Vecchio M, Craig JC, Tonelli M, Johnson DW, Nicolucci A, Pellegrini F, Saglimbene V, Logroscino G, Fishbane S, Strippoli GFM (2013). Prevalence of depression in chronic kidney disease: systematic review and meta-analysis of observational studies. Kidney Int.

[20] Mujais SK, Story K, Brouillette J, Takano T, Soroka S, Franek C, Mendelssohn D, Finkelstein FO (2009). Health-related quality of life in CKD patients: correlates and evolution over time. Clin J Am Soc Nephrol.

[21] Pagels AA, Söderkvist BK, Medin C, Hylander B, Heiwe S (2012). Health-related quality of life in different stages of chronic kidney disease and at initiation of dialysis treatment. Health Qual Life Outcomes.

[22] Juergensen E, Wuerth D, Finkelstein SH, Juergensen PH, Bekui A, Finkelstein FO (2006). Hemodialysis and peritoneal dialysis: patients’ assessment of their satisfaction with therapy and the impact of the therapy on their lives. Clin J Am Soc Nephrol.

[23] Mok MMY, Liu CKM, Lam FM, Kwan LPY, Chan GCW, Ma MKM, Yap DYH, Chiu F, Choy CBY, Tang SCW, Chan TM (2019). A longitudinal study on the prevalence and risk factors for depression and anxiety, quality of life and clinical outcomes in incident peritoneal dialysis patients. Perit Dial Int.

[24] Ulusoy SI, Kal O (2019). Relationship among coping strategies, quality of life, and anxiety and depressive disorders in haemodialysis patients. Ther Apheresis Dial.

[25] Tian N, Chen N, Li PK-T (2016). Depression in dialysis patients. Curr Opin Nephrol Hypertens.

[26] Donahue S, Quinn DK, Cukor D, Kimmel PL (2021). Anxiety presentations and treatments in populations with kidney disease. Semin Nephrol.

[27] Sesso R, Rodrigues-Neto JF, Ferraz MB (2003). Impact of socioeconomic status on the quality of life in ESRD patients. Am J Kidney Dis.

[28] Guest S (2013). Hypoalbuminemia in peritoneal dialysis patients. Adv Perit Dial.

[29] Mingardi G, Cornalba L, Cortinovis E, Ruggiata R, Mosconi P, Apolone G (1999). Health-related quality of life in dialysis patients. A report from an Italian study using the SF-36 Health Survey. Nephrol Dial Transplant.

[30] Mittal S, Ahern L, Flaster E, Mittal VS, Maesaka J, Fishbane S (2001). Self-assessed quality of life in peritoneal dialysis patients. Am J Nephrol.

[31] Jhamb M, Pike F, Ramer S, Argyropoulos C, Steel J, Dew MA, Weisbord SD, Wessfield L, Unruh M (2011). Impact of fatigue on outcomes in the hemodialysis (HEMO) study. Am J Nephrol.

[32] Cameron J, Whiteside C, Katz J, Devins GM (2000). Differences in quality of life across renal replacement therapies: a meta-analytic comparison. Am J Kidney Dis.

[33] Wu AW, Fink NE, Marsh-Manzi JVR, Meyer KB, Finkelstein FO, Chapman MM, Power NR (2004). Changes in quality of life during hemodialysis and peritoneal dialysis treatment: generic and disease specific measures. J Am Soc Nephrol.

[34] Aguiar R, Pei M, Qureshi AR, Lindholm B (2018). Health-related quality of life in peritoneal dialysis patients: a narrative review. Semin Dial.

