# Supplementary material for: Comparison of quality of life in patients with advanced chronic kidney disease undergoing haemodialysis, peritoneal dialysis and conservative management in Johannesburg, South Africa: a cross-sectional, descriptive study
Source: BMC Psychol. 2023 May 8;11:151. doi: 10.1186/s40359-023-01196-1 (PMC10165796; doi:10.1186/s40359-023-01196-1)
Supplement: Supplementary file 1 — Additional file 1. Multicollinearity analysis for included regression parameters [file 40359_2023_1196_MOESM1_ESM.docx]

| **Supplementary table S1. Multicollinearity analysis for included regression parameters** | | |
| --- | --- | --- |
| ***Patients living with advanced chronic kidney disease*** | | |
| **Parameter** | **VIF** | **Tolerance** |
| Age | 1.50 | 0.67 |
| Sex (male, female) | 1.17 | 0.86 |
| Ethnicity (Black African, Non-Black African) | 1.06 | 0.94 |
| Relationship status (married, partner, no relationship) | 1.09 | 0.92 |
| Source of income (employed, social grant recipient, no personal source of income) | 1.11 | 0.90 |
| Diabetes (diabetic, not diabetic) | 1.29 | 0.77 |
| On dialysis programme (on either haemodialysis or peritoneal dialysis, conservative management) | 1.66 | 0.60 |
| Haemoglobin | 1.14 | 0.88 |
| Phosphate | 1.18 | 0.85 |
| Parathyroid hormone | 1.25 | 0.80 |
| Albumin | 1.20 | 0.83 |
| Mean | 1.24 |  |
| ***Patients receiving dialysis*** | | |
| Sex (male, female) | 1.13 | 0.89 |
| Ethnicity (Black African, Non-Black African) | 1.04 | 0.96 |
| Relationship status (married, partner, no relationship) | 1.05 | 0.95 |
| Source of income (employed, social grant recipient, no personal source of income) | 1.08 | 0.93 |
| Prescribed peritoneal dialysis (prescribed PD, prescribed HD) | 1.64 | 0.61 |
| Dialysis vintage | 1.24 | 0.81 |
| Haemoglobin | 1.37 | 0.73 |
| Parathyroid hormone | 1.20 | 0.83 |
| Albumin | 1.36 | 0.73 |
| Mean | 1.24 |  |
