# Supplementary figures and images for: Comparison of quality of life in patients with advanced chronic kidney disease undergoing haemodialysis, peritoneal dialysis and conservative management in Johannesburg, South Africa: a cross-sectional, descriptive study
Source: BMC Psychol. 2023 May 8;11:151. doi: 10.1186/s40359-023-01196-1 (PMC10165796; doi:10.1186/s40359-023-01196-1)

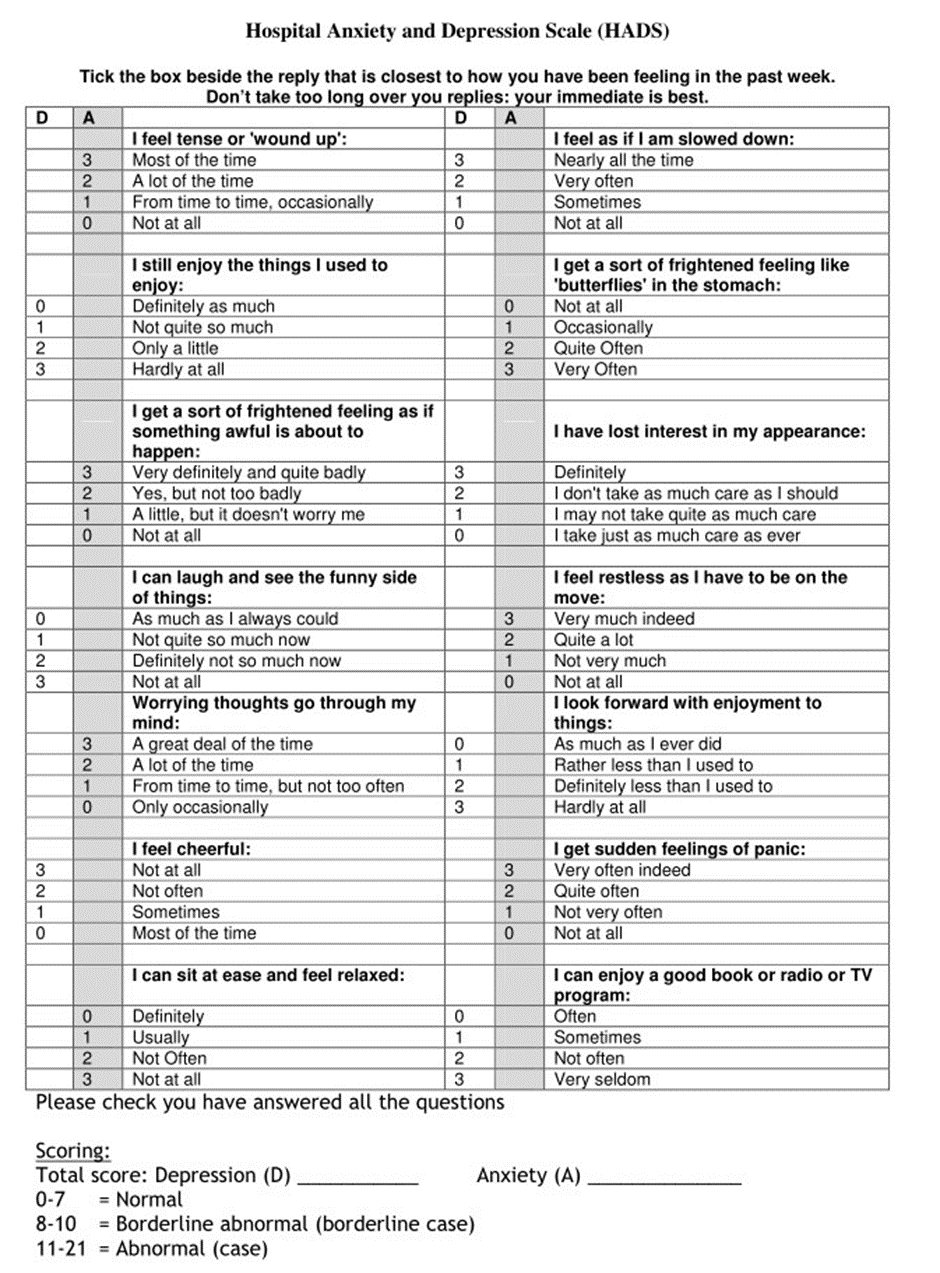

Supplement: Supplementary file 2 — Additional file 2. Hospital Anxiety and Depression Scale [file 40359_2023_1196_MOESM2_ESM.docx]

**Supplementary data: KDQOL-36 survey**

**
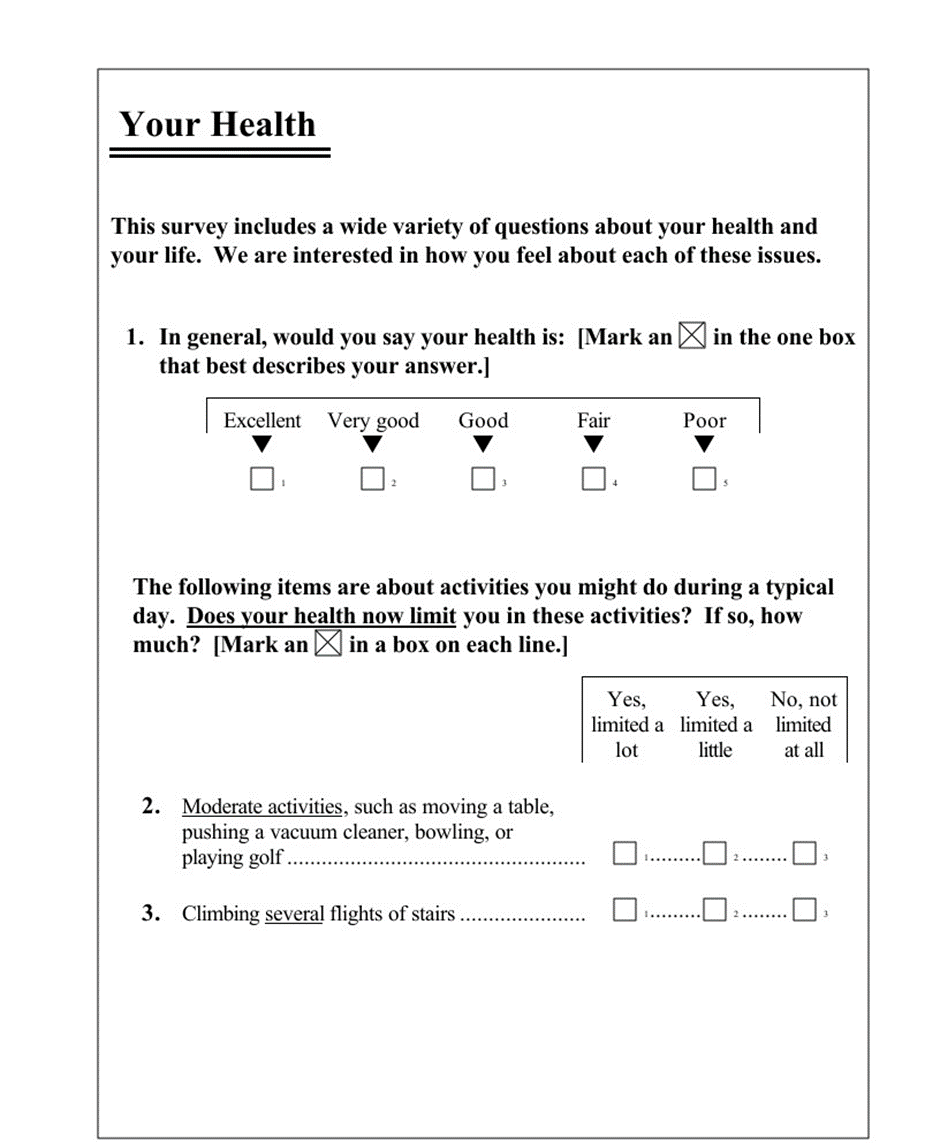
**


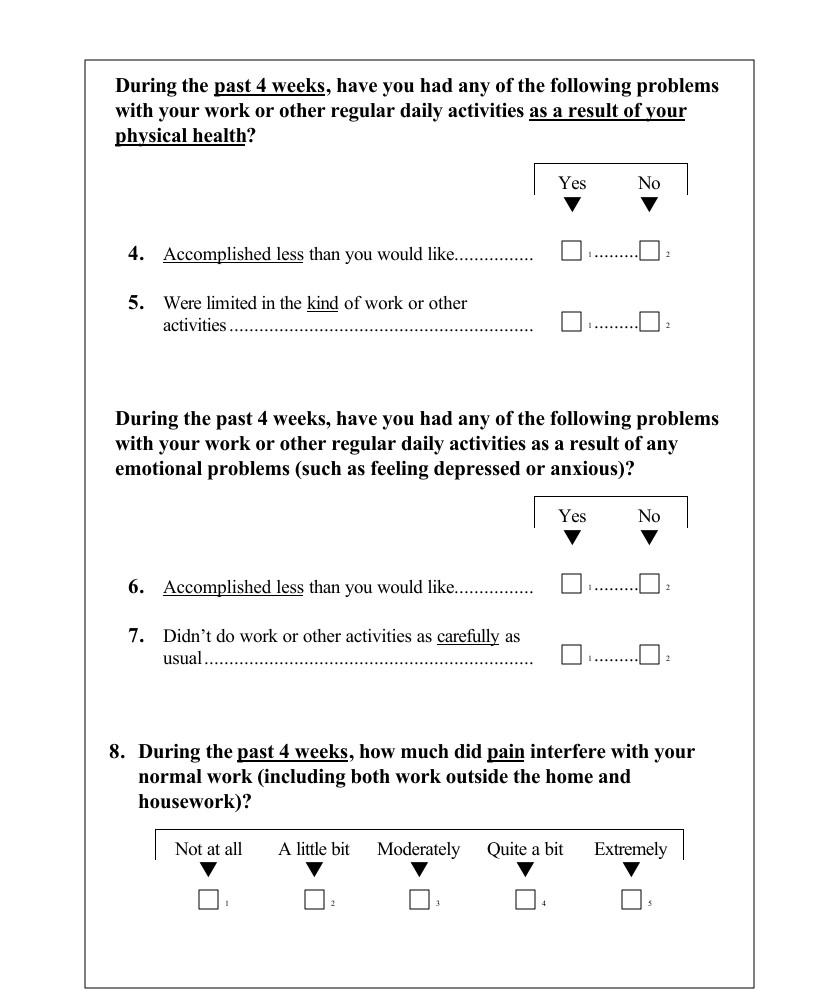


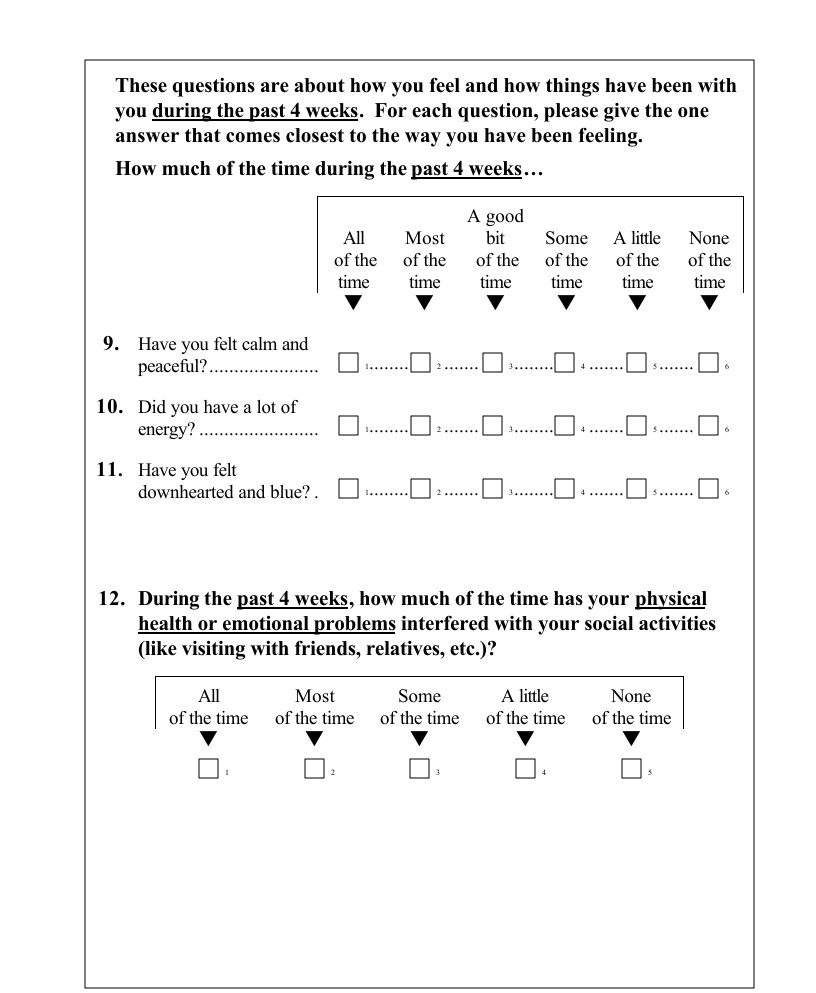


**
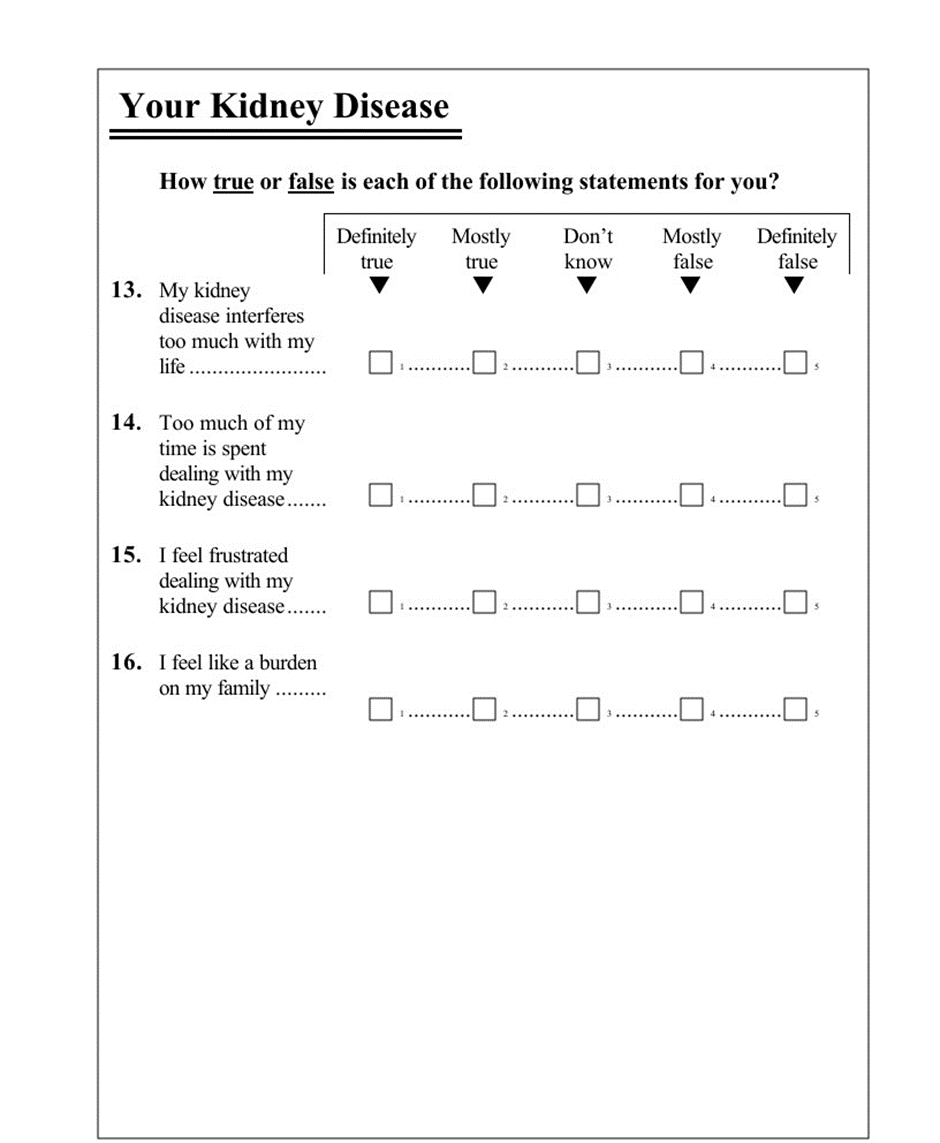
**

**
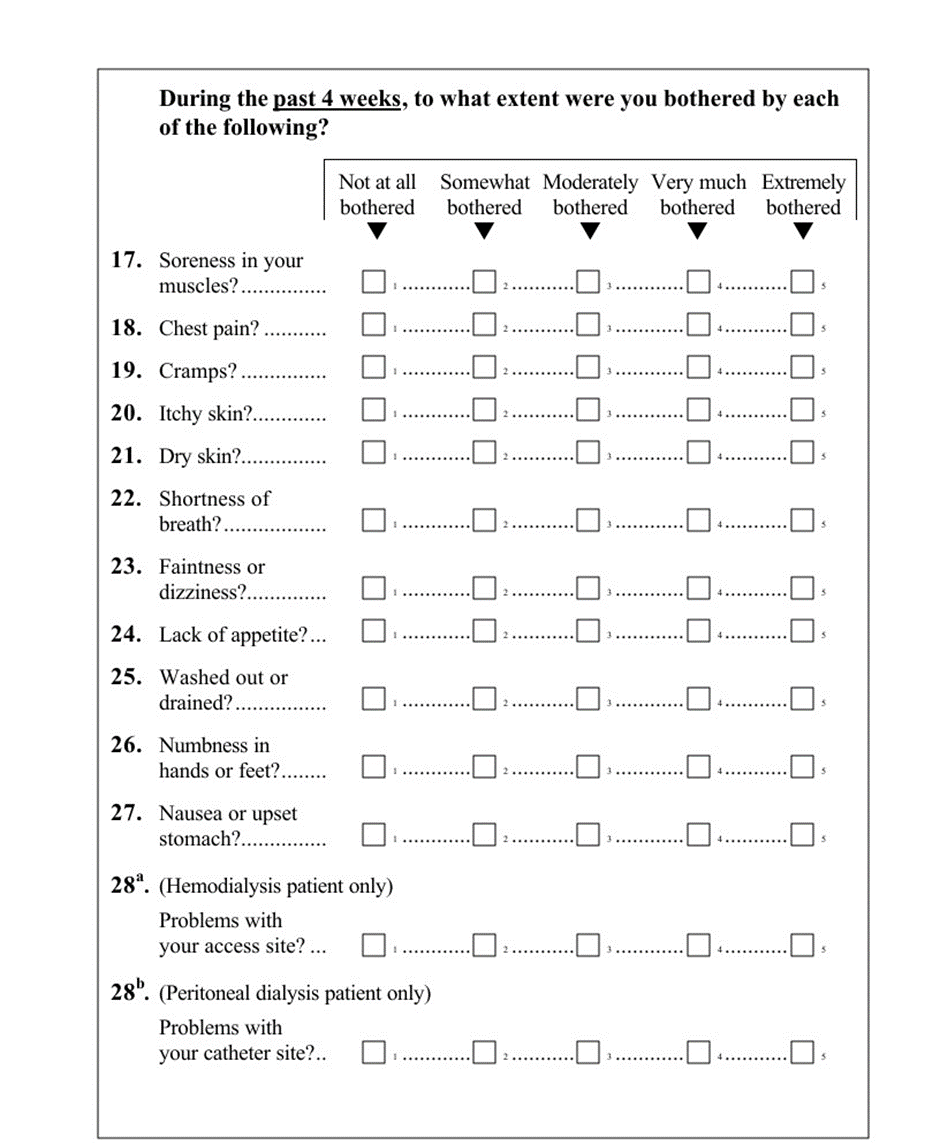
**

**
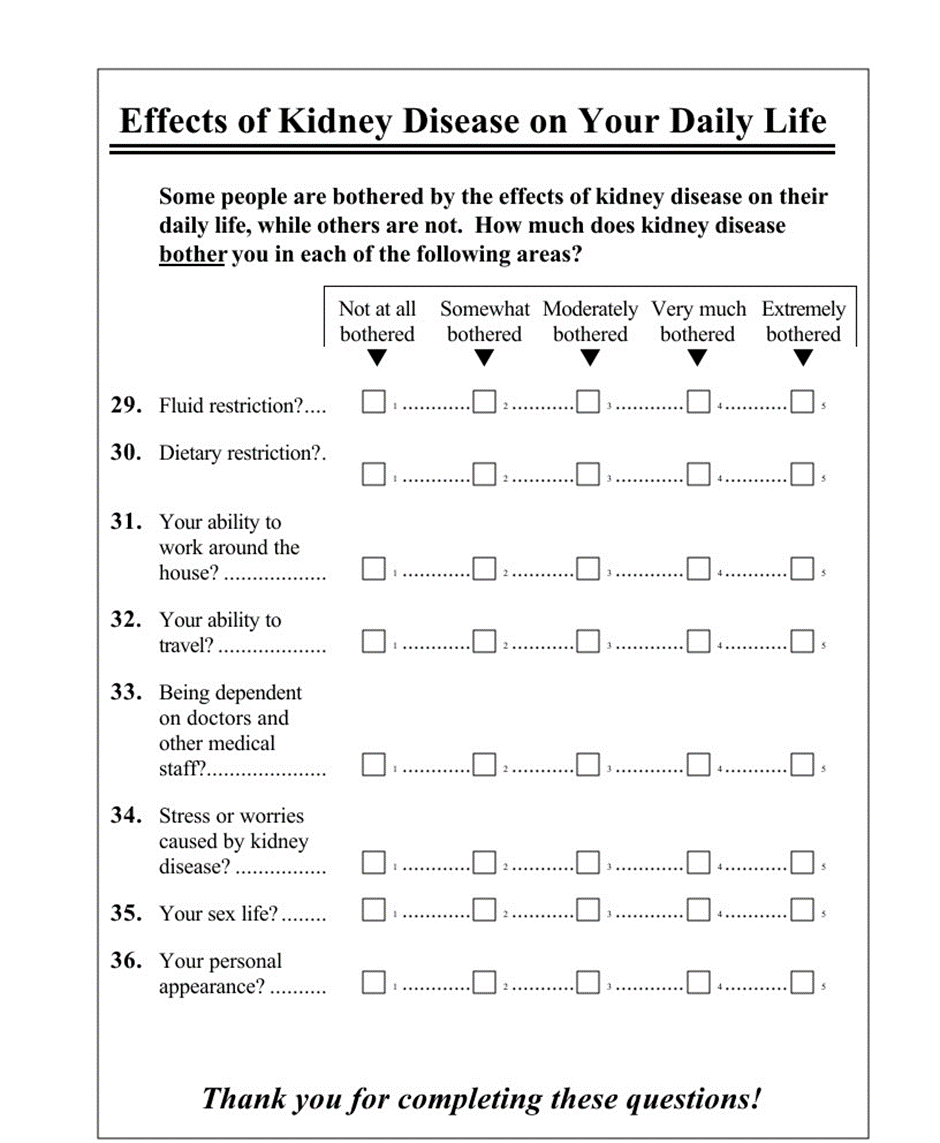
**

Supplement: Supplementary file 3 — Additional file 3. KDQOL-36 Survey [file 40359_2023_1196_MOESM3_ESM.docx]
